# Zoonotic implications of camel diseases in Iran

**DOI:** 10.1002/vms3.239

**Published:** 2020-03-11

**Authors:** Roya Mohammadpour, Mohsen Champour, Fateh Tuteja, Ehsan Mostafavi

**Affiliations:** ^1^ Department of Epidemiology and Biostatistics Research Centre for Emerging and Reemerging infectious diseases Pasteur Institute of Iran Tehran Iran; ^2^ Department of Clinical Sciences School of Veterinary Medicine Ferdowsi University of Mashhad Mashhad Iran; ^3^ National Research Centre on Camel Bikaner Rajasthan India

**Keywords:** dromedary camel, epidemiology, Middle East, zoonoses

## Abstract

Approximately 60% of all human pathogens and 75% of emerging infectious diseases are zoonotic (of animal origin). Camel zoonotic diseases can be encountered in all camel‐rearing countries. In this article, all studies carried out on camel zoonotic diseases in Iran are reviewed to show the importance of camels for public health in this country. More than 900 published documents were systematically searched to find relevant studies from 1,890 until late 2018. The collected articles were classified according to the aetiological agents. In this study, 19 important zoonotic diseases were reported among Iranian camels including listeriosis, leptospirosis, plague, Q fever, brucellosis, campylobacteriosis, tuberculosis, pasteurellosis, clostridiosis, salmonellosis, *Escherichia coli* infections, rabies, camelpox, Middle East respiratory syndrome coronavirus, Crimean‐Congo haemorrhagic fever, echinococcosis, cryptosporidiosis, toxoplasmosis and dermatophytosis, most of which belong to bacterial, viral, parasitic and fungal pathogens, respectively. Results show that camels are one of the most important sources of infections and diseases in human; therefore, continuous monitoring and inspection programs are necessary to prevent the outbreak of zoonotic diseases caused by this animal in humans.

## Graphical abstract

The importance of zoonotic disease is not hidden, and these diseases have had a tremendous effect on human population throughout history as more than 60% of human diseases are zoonotic. Also the importance of camel farming as a source of milk and meat in world market and especially in the Middle East is developing. So camel zoonotic disease can have a significant role in public health in all around the world including Iran. Indirect zoonoses by cross transmission of camel diseases through other livestock or vice versa do require attention.

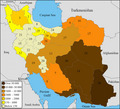

## 1 INTRODUCTION

A zoonosis is any disease or infection that is naturally transmissible from vertebrate animals to humans. More than 60% of human infectious diseases are caused by zoonotic pathogens which have been responsible for some of the most fatal diseases such as Ebola and severe acute respiratory syndrome (SARS) in recent years (Belay et al., 2017). Zoonotic diseases may be obtained or transmitted in a variety of ways including direct contact, through the air (aerosol), contact with an inanimate object that harbours the disease (fomite transmission), oral ingestion and arthropod bite (Hersom, Irsik, & Thrift, 2008). According to studies, camels are one of the important carriers and sources of infection for human, livestock and wildlife in Iran and other parts of the world where camel lives. Some camel diseases and infections can pose a significant threat to public health, including Middle East respiratory syndrome coronavirus (MERS‐CoV; Alkhamis et al., 2018). The first case of this disease was reported in Saudi Arabia in 2012. Subsequently, the disease caused more than 1,500 confirmed human infections with over 580 deaths (Alkhamis et al., 2018). Several studies implicated that dromedary camel is the primary intermediate host and the source of zoonotic inaugurations (Du & Han, 2016). The most important camel zoonotic diseases and infections reported worldwide are actinomycosis (Kilic & Kirkan, 2004), anthrax (Musa, Shomein, Abd el Razig, Meki, & Hassan, 1993), borreliosis (Helmy, 2000), chlamydiosis (Elzlitne & Elhafi, 2016), clostridial diseases (Wernery, Ul‐Haq, Joseph, & Kinne, 2004; Younan & Gluecks, 2007), balantidiasis (Tajik, Farda, paidar, Anousheh, & Dehghani, 2013), melioidosis (Bergin & Torenbeeck, 1991), *Staphylococcus aureus* (Jaradat, Al Aboudi, Shatnawi, & Ababneh, 2013), *Corynebacterium ulcerance* (Tejedor, Martin, Lupiola, & Gutierrez, 2000), mycoplasma (Mederos‐Iriarte et al., 2014), streptococcal (Heller, Anderson, & Silveira, 1998), trypanosomiasis (Bennoune, Adili, Amri, Bennecib, & Ayachi, 2013), fascioliasis (Haridy & Morsy, 2000), schistosomiasis (Singh, Borah, Dadhich, & Sharma, 2013), sarcopticosis (Sk, Tuteja, & Sena, 2009), hepatitis(Woo et al., 2014), influenza A virus (Yamnikova et al., 1993), rift valley fever (Swai & Sindato, 2015), West Nile fever (El‐Harrak et al., 2011), leptospirosis, plague, Q fever, brucellosis, campylobacteriosis, tuberculosis, pasteurellosis, clostridiosis, salmonellosis, *Escherichia coli*, glanders, rabies, camelpox, Middle East respiratory syndrome coronavirus (MERS‐CoV) infection, Crimean‐Congo haemorrhagic fever (CCHF), cysticercosis, toxocariasis, echinococcosis, giardiasis, surra, leishmaniasis, trichinellosis, cryptosporidiosis, toxoplasmosis and dermatophytosis (Wernery, Kinne, & Schuster, 2014). Some of these diseases are very common in camels, while others are rare. Some of these pathogens cause clinical diseases, whereas others are subclinical.

The ability of camels to survive in arid and semiarid areas of the world, endurance in prolonged drought and above all, a high potential to convert the scant resources of the desert into milk and meat, making them more important for raising in order to compensate for more food demand in the future because of globally growing human population and climate change (Al‐Jassim & Sejian, 2015). Camel raising is not only socially acceptable but also economically relevant; therefore, authorities of camel farming are focusing on increasing camel number and, consequently, the farmer income (Mirzaei, 2012). In this situation, control of camel pathogens will continue to be a highly important component of efficient food production and become associated more overtly with the food security agenda (Al‐Jassim & Sejian, 2015). In some regions, camel milk and liver are consumed raw without any heat treatment. There are reports on human plague outbreaks due to eating raw camel liver and meat in Libya, Saudi Arabia, Jordan and Afghanistan in 1976, 1994, 1997 and 2007, respectively (Leslie et al., 2011). In addition, there is close contact between a herdsman and camels on several occasions during watering, riding, grooming and milking. For instance, senile, debilitated or sick animals are often well‐nursed and hand‐fed for long periods (Abbas, Zubeir, & Yassin, 1987); therefore, it may increase the contact between the animal and human and contribute to the transmission of some zoonotic diseases as well. In countries raising camels (e.g. Iran), there is considerable direct contact between farmers and camels, and also meat and milk consumption, which are among the sources of infection. Improper importation or smuggling of camels from neighbouring countries, such as Afghanistan, Pakistan and the United Arab Emirates (UAE), can also account for a source of new zoonotic diseases import.

Despite the huge and rising impacts of zoonotic diseases on human health, there are still gaps in our knowledge of how some zoonotic infections develop and spread in different populations. Accordingly, gathering this information can be helpful to predict and prevent future outbreaks. Public health authorities should focus on detection, investigation and control of these threats with a health‐based approach.

**Figure 1:**
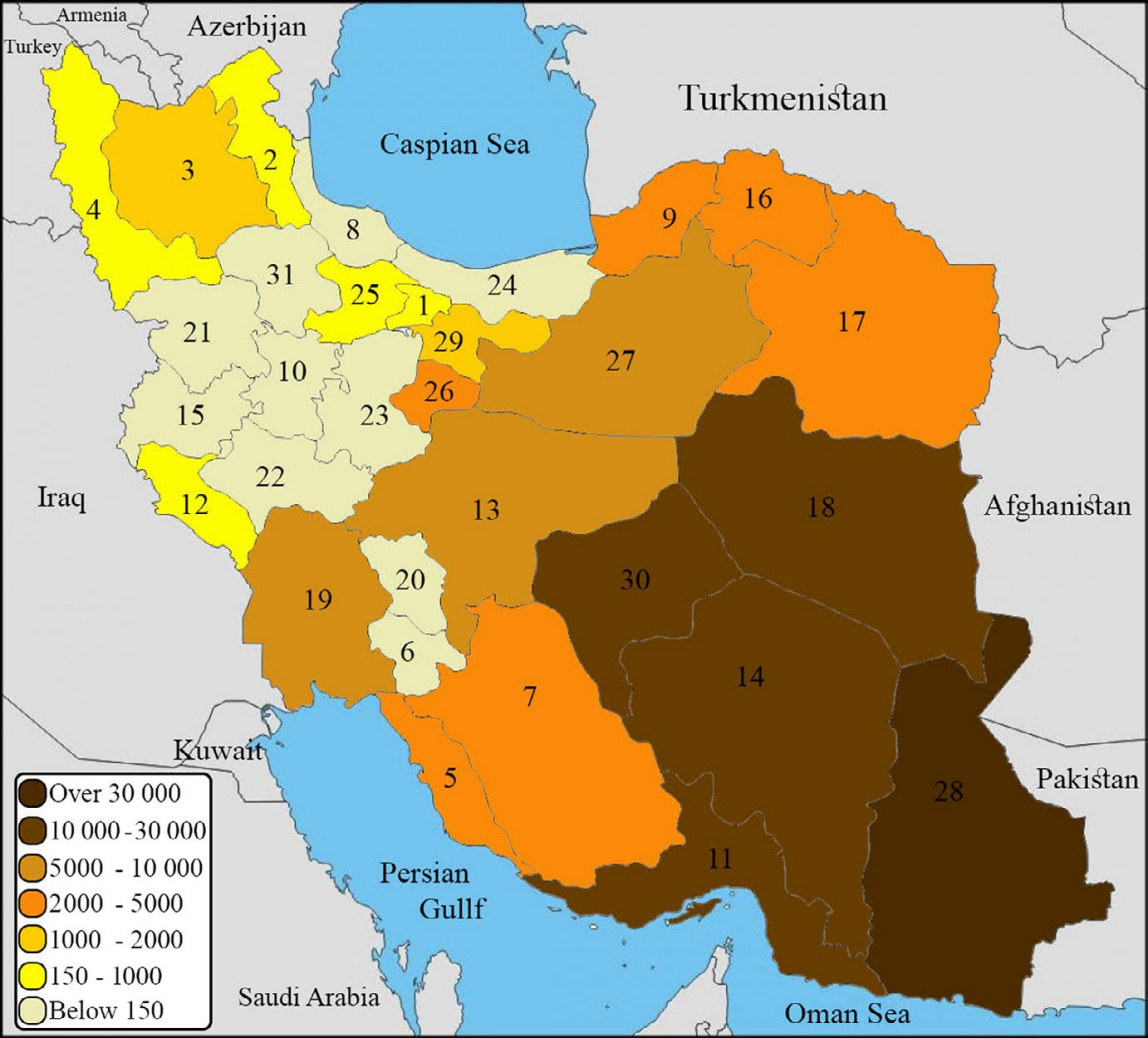
Iranian camel's numbers categorized by provinces according to the census of Ministry of Jihad –e‐ Agriculture, 2017 (Ebadzadeh et al., 2018). (1) Alborz (2) Ardabil (3) Azerbaijan, East (4) Azerbaijan, West (5) Bushehr (6) Chahar Mahaal and Bakhtiari (7) Fars (8) Gilan (9) Golestan (10) Hamadan (11) Hormozgān (12) Ilam (13) Isfahan (14) Kerman (15) Kermanshah (16) Khorasan North (17) Khorasan, Razavi (18) Khorasan South (19) Khuzestan (20) Kohgiluyeh and Boyer‐Ahmad (21) Kurdistan (22) Lorestan (23) Markazi (24) Mazandaran (25) Qazvin (26) Qom (27) Semnan (28) Sistan and Baluchestan (29) Tehran (30) Yazd (31) Zanjan

Since the use of camel products in Iran has increased in recent years, and the movement of these livestock between the provinces and neighbouring countries is carried out without special restrictions, these animals and their products can be a source of transmission of some diseases to human (Figure 1). Hence, having more information about the health status of camels is important for health stakeholders and healthcare providers in the country. In this study, the camel zoonotic diseases and infections in Iran are outlined by focusing on the aetiology of diseases and infections, clinical signs, methods of diagnosis, routes of transmission and determining their distribution pattern in order to show camel's public health importance in Iran. It is expected that information from this study be used by relevant authorities in the field of medicine and veterinary medicine.

## 2 MATERIALS AND METHODS

### 2.1 Search strategy

To review relevant studies, Medline (PubMed), ISI Web of Knowledge, Science Direct, Embase, Scopus and Google Scholar were systematically searched to find all publications from Iran using the keywords of "camel" and "Iran". The retrieved papers that reported camel zoonotic diseases with major public health importance were included in the study.

More than 900 published documents were systematically searched to find related studies from 1890 until late 2018. The included papers were written in English, French and Persian. Iranian search engines such as Scientific Information Database (SID), MEDLIB, Magiran, IranMedex and proceeding of the first national congress of camel were also searched to find the related papers.

Moreover, in order to maximize the sensitivity of the search, bibliographies of the identified studies were screened for additional relevant studies. All the resultant titles and abstracts for the given disease were included in the review. In addition, this review assessed all full‐text articles and incorporated related documents. The collected studies were classified according to the infectious agent (viral, bacterial, parasitic and fungal diseases).

Two authors (RM and HM) independently screened the title and abstract of all obtained studies and then reviewed the full texts of the retrieved studies that met research criteria. The authors were not blinded to the names of the studies' authors and journals. All disagreements between the authors about the final selection of studies were resolved by negotiation with a third author (CH). The agreement rate of the two authors was more than 90%. The variables that were extracted for data analysis included: study design, year and location of the study, disease type, transmission method and the signs of disease in camel.

Six items from the Strengthening the Reporting of Observational Studies in Epidemiology (STROBE) statement checklist were selected and used for assessing the quality of reporting (Vandenbroucke et al., 2007).

## 3 RESULTS

This review covers 19 important zoonotic diseases reported in camels of Iran, most of which belonged to bacterial, viral, parasitic and fungal pathogens in order of prevalence.

### 3.1 Bacterial zoonotic diseases

In this study, listeriosis, leptospirosis, plague, Q fever, brucellosis, campylobacteriosis, tuberculosis, pasteurellosis, clostridiosis, salmonellosis and *E*. *coli* infections were reported among the camels (Table 1).

**Table 1:** Summary of zoonotic bacterial diseases of camels reported in Iran; 1890–2018

A. Bacterial diseases					
Disease	Year	Location	Techniques	No. positive/ No. tested (%)	Ref
Leptospirosis	1959	Various area of Iran	–	1/5 (20%)	(Rafyi & Maghami, 1959)
	1996	South Khorasan (Tabas)	Serological test	1/14 (7%)	(Hadian, 1996)
	2008–2011	Yazd	Microscopic agglutination test	22/8% No Numeral data	(Sazmand, 2012)
	2010–2011	Various area of Iran	Bacterial culture, multiplex PCR	7/35 (20%), 8/35 (22.85%)	(Safarpoor Dehkordi et al., 2012; Safarpoor Dehkordi & Taghizadeh 2012)
	2012	Isfahan	PCR	19/130 (14.61%)	(Doosti et al., 2012)
	2012	Isfahan	Bacterial culture, Multiplex PCR	7/49 (14.29%), 8/49 (6.33%)	(Dehkordi et al., 2012; Dehkordi and Taghizadeh 2012)
	2013	Yazd	Microscopic agglutination test	30/128 (32.4%)	(Hajikolaei et al., 2013)
	2013	Qom	Microscopic agglutination test	51/183 (27.87%)	(Talebi, 2007)
	2014	Ardabil	Microscopic agglutination test	12/60 (20%)	(Afkhamnia, Avagyan, Khaki, Bidhendi, & Mostafaey, 2014)
Q fever	1959	–	ELISA	2/12 (16.66%)	(Moghaddas, 2012 2012)
	2011	Isfahan	PCR	14/130 (10.76%)	(Doosti et al., 2014)
	2015	North, South and Razavi Khorasan	ELISA	48/168 (28.7%)	(Janati Pirouz et al., 2015)
	2016	Khorasan ( North, South, Razavi)	PCR	4/167 (2.4%)	(Pirouz, Mohammadi, Mehrzad, & Aziz zadeh, 2016)
Brucellosis	1987	Fars	Tube agglutination, RBPT	2/238 (0.84%)	(Motamedi, 1987)
	1986–1987	Qazvin	RBPT, SAT, 2ME, CFT	77/935 (8%)	(Zowghi & Ebadi, 1991)
	1994	Isfahan (Najaf Abad)	RBPT	5/100 (0.05%)	(Miranzade, 1994)
	1999	Bushehr	RBPT, SAT, 2ME, CFT	5/258 (1.93%)	(Khadjeh, Zowghi, & Zarif‐fard, 1999)
	2005	Isfahan (Najaf Abad)	RBPT, Wright, 2ME	11/384 (2.84%)	(Pourjafar et al. 2005)
	2007	Hormozgan	RBPT	3/103 (2.91%)	(Garib, 2011)
	2011	Sistan and Baluchestan	RBPT, SAT, 2ME	17/500 (3.4%)	(Sargaz, 2011)
	2008–2011	Yazd	RBPT	149/395 (37.83%)	(Sazmand, Rasooli, et al., 2012)
	2012	Isfahan (Najaf Abad)	(RBPT, mRB, Wright, 2ME), PCR, Culture	39/310 (12.58%) mRB; 27/310 (8.71%) RBPT; 7/310 (2.26%) Wright; 6/310 (1.94%) 2ME; 18.6/310 (6%) PCR	(Ghorbani et al., 2013)
	2012	Isfahan	Multiplex PCR, Culture	4/35 (11.42%) aborted fetus	(Dehkordi et al., 2012; Dehkordi and Taghizadeh 2012)
	2012	Various part of Iran	Conventional PCR, real‐time PCR assays	201/618 (32.52%) C‐PCR, 143/201 (71%), RT‐PCR	(Dehkordi et al., 2012; Dehkordi and Taghizadeh 2012)
	2014	Isfahan	PCR, blood sample, lymph node	14/123 (11.38%) blood sample; 16/123 (13.01%) lymph node samples	(Khamesipour, Doosti, Mobarakeh, & Komba, 2014; Khamesipour, Rahimi, Shakerian, Doosti, & Momtaz, 2014)
	2015	Isfahan (Najaf Abad)	RBPT, Tube agglutination, 2ME, PCR	18/150 (12%) RBPT; 12/150 (8%) TG, 9/150 (6%) 2ME; 3/150 (1.3%) PCR	(Mahzunieh & Salami, 2015)
Campylobacteriosis	2006–2006	Fars, Bushehr	Culture PreT‐KB method	3/145 (2%)	(Baserisalehi et al., 2007)
	2007–2008	Isfahan (Najaf‐Abad)	Bacteriological examination	5/94 (2%)	(Rahimi, Momtaz, & Nozarpour, 2010)
	2008–2009	Isfahan, Yazd	Microbiological analysis	1/107 (0.9%)	(Rahimi, Momtaz, et al., 2010)
	2010	Chaharmahal & Bakhtiari, Khuzestan	PCR	3/130 (2.3%)	(Rahimi et al., 2013)
Plague	1960	Iran	–	–	(McGrane & Higgins, 1985)
	1974	Iran	–	–	(Fedorov, 1960)
Tuberculosis	–	Khorasan Razavi	PCR	4/100 (4%)	(Hashemi et al., 2002)
	2009–2010	Khorasan Razavi (Mashhad)	PCR	17/102 (16.66%)	(Soleymani Babadi et al., 2012)
Pasteurellosis	2010	Fars (Larestan)	Morphological, cultural, biomedical characterization	53/100 (53%)	(Esmaeili et al., 2010)
	2014	Iran	DNA extraction, PCR assay	104/971 (10.7%)	(Chitgar et al., 2014)
	2014–2015	Fars (Larestan)	Morphological, cultural, biomedical characterization	4/5 (80%) dead camels, 31/48 (64.58%) clinical camels, 8/109 (7.34%) healthy camels	(Tahamtan et al., 2017)
Salmonellosis	1992	North East of Iran	Bacteriological examination Serological test	14/113 (12.39%), 38/102 (37.25)	(Moghaddas, 2012 2012)
	1994	Isfahan (Najaf Abad)	Agar gel immunodiffusion test (AGIT)	1/59 (1.69%)	(Miranzade, 1994)
	2007–2008	Isfahan (Najaf Abad)	Bacteriological examination	Case report	(Rahimi et al., 2012)
	2010	Isfahan (Najaf Abad)	Agar gel immunodiffusion test	3/384 (0.78%)	(Pourjafar, Mahzounieh, Zahraei Salehi, & Habibollahi Khorasgani, 2010)
	2010	Tehran	Microbiology test, Histopathology, Gross Pathology, Clinical Pathology	1/94 (1.1%)	(Nour‐Mohammadzadeh et al., 2010)
	2010–2011	West Azerbaijan (Urmia region)	Bacteriological examination, PCR	4/100 (4%)	(Ahmadi, 2012)
	2011–2012	Fars and Khuzestan	Bacteriological examination, PCR	1/50 (2.0%)	(Rahimi et al., 2012)
	2012	Razavi Khorasan	Microbiology	10%	(Gholami et al., 2014)
	2012	Various parts of Iran	Microbiology	84/196 (43%)	(Sepehr, 2012)
	2013	Kerman	PCR	8%	(Zavarshani, Kownani, Estabraghi, & Yarahmadi, 2015)
	2015	Golestan	Quantitative agglutination test	9/9 (100%)	(Rabbani Khorasani, Eatemadifar, Azadbakht, Emami, & Khormali, 2015)
	2016	Razavi Khorasan (Mashhad)	Microbiology	2/10 (20%)	(Mohammady & Najafi Mosleh, 2017)
Listeriosis	2007–2008	Isfahan Najaf Abad	Bacteriological test	9/94 (9.6%)	(Rahimi, Momtaz, et al., 2010)
	2011	Sistan and Baluchestan (Sistan)	Bacteriological examination	3/80 (3.75%)	(Safdari & Jahantigh, 2014)
	2011	Various parts of Iran	Culture, PCR, Technique Real‐Time PCR,	6/101 (5.94%) Milk; 3/100 (3%) Faeces; 10/79 (13.92%) Vaginal Swab; 8/95 (9.47%) Urine	(Dehkordi, Barati, Momtaz, Ahari, & Dehkordi, 2013; Dehkordi, Haghighi Borujeni, Rahimi, & Abdizadeh, 2013)
	2011–2012	Tehran	Microbiological tests	6/24 (25%) Frozen meat, 12/24 (50%) Fresh meat	(Mashak et al., 2015)

#### 3.1.1 Leptospirosis

Leptospirosis is a disease that causes intensive clinical illness in humans and animals. The causative agent develops directly within its hosts and indirectly in the environment. More than 250 serovars of *Leptospira* are recognized as pathogenic agents (Ellis, 2015; Ningal et al., 2015). Leptospirosis has been reported from all parts of the world but it is more common in tropical and subtropical areas with high rainfall periods. Annually, about 7–10 million people are infected by *Leptospira* spp. worldwide (Hartskeerl, Collares‐Pereira, & Ellis, 2011).

Leptospirosis is a disease with clinical manifestations such as stillbirth, abortion, haematuria, infertility and death in animals (McGrane & Higgins, 1985; Wernery & Kaaden, 2002). Serological evidence of camel leptospirosis was reported from Iran's neighbouring countries such as Saudi Arabia, UAE, Afghanistan and the former USSR (Hussein & El Nabi, 2009). Leptospirosis may be more important in camels because there are increasing tendency towards camel meat and dairy products in Iran and other neighbouring countries (Doosti, Ahmadi, & Arshi, 2012).

Transmission of leptospirosis to human can occur via several ways such as contact with soil or water contaminated with the urine of infected animals and consumption of unpasteurized milk and dairy products, and people with direct contact (e.g. veterinarians, farmers and abattoir workers) are at high risk of infection (Ningal et al., 2015).

The disease is more prevalent in northern Iran (Rafiei, Hedayati Zadeh Omran, Babamahmoodi, Alizqadeh Navaee, & Valadan, 2012). Leptospirosis in camel was first reported in Iran in 1959. In that report, 20% of serum samples were positive for *Leptospira icterohemorrhagic* serotype (Hajikolaei, Sazmand, Abdollahpour, & Moghadam, 2013; Mustafa, 1987; Rafyi & Maghami, 1959). In 1996, 41 camels showed signs of recurrent fever, anorexia, severe constipation and jaundice in Tabas (northeastern Iran), and finally, 78% of camels died and 7.14% of serum samples were positive for *L. canicola* (Hadian, 1996). From 2008 to 2014, leptospiral infection varied from 20% to 32.4% by microscopic agglutination test (Sazmand, 2012), 6.33% to 22.85% by PCR (Doosti et al., 2012) and 14.29% to 20% in bacterial cultures (Safarpoor Dehkordi, Saberian, & Momtaz, 2012; Safarpoor Dehkordi and Taghizadeh 2012) in camels studied from different regions of the country (Table 1). In two serological studies on *Leptospira* spp. infection in 2013, 2.34% and 27.87% of serum samples from camels of Yazd and Qom provinces, respectively, were infected with at least one of *Leptospira* spp. serotypes. Among positive sera, *L. Pomona* (57.9%), *L. canicola* (23.7%), *L. hardjo* (10.5%), *L. grippotyphosa* (5.3%) and *L. icterohaemorrhagiae* (2.6%) were the most frequent serovars (Hajikolaei et al., 2013).

**Figure 2:**
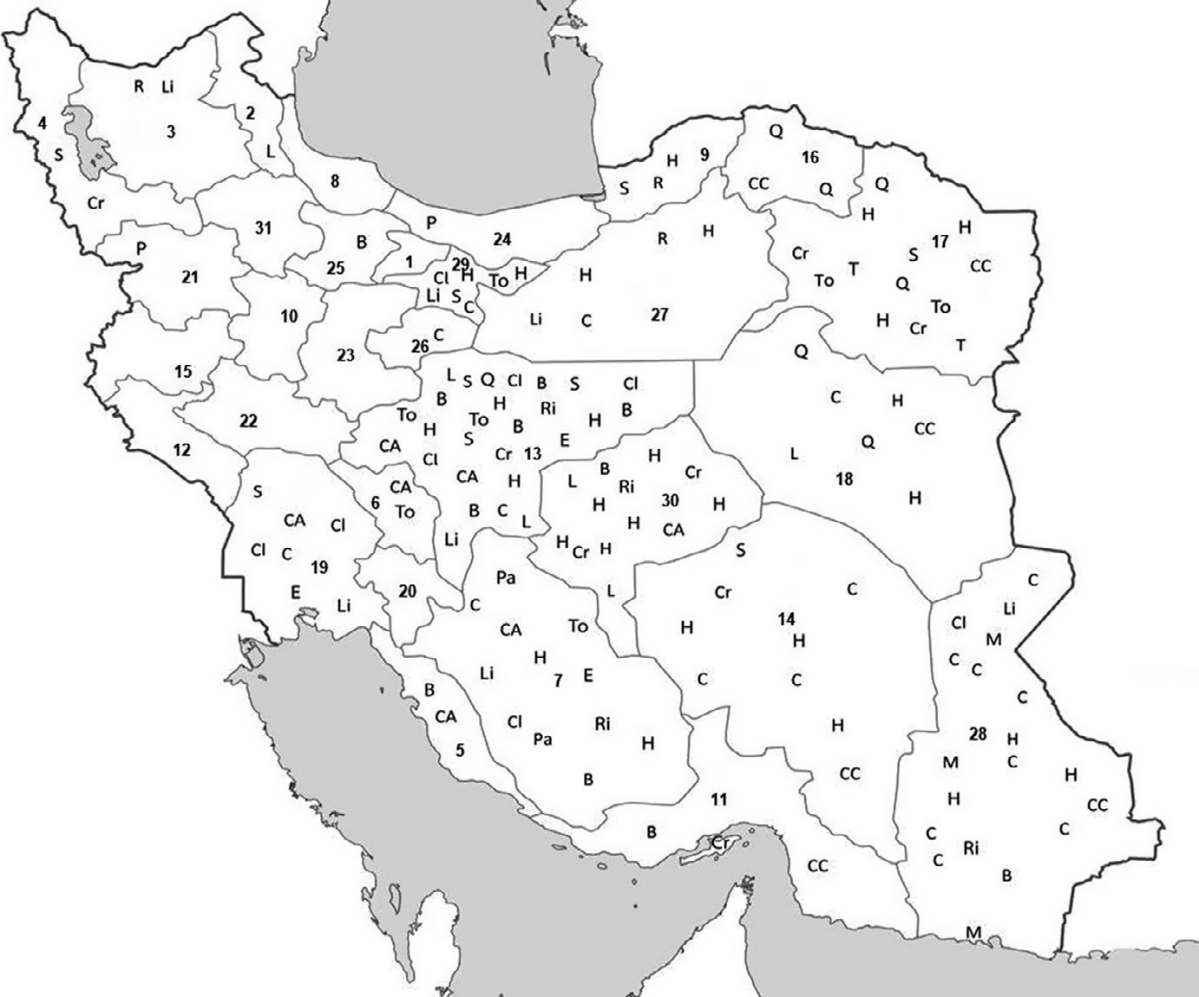
Distribution of camel zoonotic disease in various parts of Iran. Including 22 provinces of report. Abbreviations: B, Brucellosis; C, Camelpox; CA, Campylobacteriosis; CC, Crimean‐Congo Haemorrhagic Fever; Cr, *Cryptosporidium* spp.; H, Hydatidosis; L, Leptospirosis; Li, Listeriosis; M, MERS‐CoV; P, Plague; Pa, Pasteurellosis; Q, Q fever; R, Rabies; S, Salmonellosis; T, Tuberculosis; To, Toxoplasmosis

According to reports on camel leptospirosis, there is a necessity to further study this disease to figure out the clinical signs and its importance in transmitting diseases to humans in Iran and all over the world (Figure 2).

#### 3.1.2 Q fever

Q fever is a highly contagious zoonotic disease caused by *Coxiella burnetii*. A large number of animal species including wild and domestic mammals, birds. If not, please provide clear guidance on where it should be cited in the text. and arthropods, such as ticks, contribute to the transmission of *C. burnetii* (Maurin & Raoult, 1999). Cattle, sheep, camel and goat are the main sources of the infection (Doosti, Arshi, & Sadeghi, 2014).

Q fever has been reported in all over the world (Angelakis & Raoult, 2010), and recently in the neighbouring countries of Iran, including Oman, Iraq, Afghanistan, UAE, Turkey and Saudi Arabia (Mostafavi, Rastad, & Khalili, 2012). *Coxiella burnetii* is one of the most widespread infections in camels. Q fever in camel is reported nearly from all parts of North and East Africa and the Middle East. A high prevalence (62%) of Q fever antibodies in camels has been reported from Saudi Arabia, Iran's neighbouring country (Hussein et al., 2015).

Infection in camels is usually subclinical but it can cause late abortion (Janati Pirouz, Mohammadi, Mehrzad, Azizzadeh, & Nazem Shirazi, 2015). Infected camels shed bacteria in urine, faeces, milk, as well as through placenta (Janati Pirouz et al., 2015).

Humans are infected mainly by inhalation of contaminated aerosols (airborne), ingestion of milk or fresh dairy products of infected animals, and exposure to placenta and occasionally ticks (Masala et al., 2004). In human, Q fever is most often asymptomatic but it can appear as an acute or chronic disease (Angelakis & Raoult, 2010; Norlander, 2000).

Q fever is an endemic disease in Iran, which is mostly reported in human, domestic animals and ticks from almost all the provinces of the country (Mostafavi et al., 2012). However, there is little information about the epidemiology of coxiellosis in camels (Janati Pirouz et al., 2015).

In seroepidemiological surveys from 1959 to 2016, samples of camel's blood positive for *C. burnetii* ranged from 0.0 to 63.6% in Khorasan and Isfahan provinces, respectively (Mogghadass, 2012). Accordingly, considerations for camel hygiene, especially in farms, and restrictions on contact with other domestic animals are important factors to reduce *C. burnetii* infection among camels.

#### 3.1.3 Brucellosis

Brucellosis is an infectious bacterial disease in human and many other animal species. It is caused by a different genus of *Brucella* (Corbel, 1997). *Brucella melitensis* is the most common cause of human disease. Sheep, goat and camel are the main sources of infection (Alavi, Mugahi, Nashibi, & Gharkholu, 2014). Annually, brucellosis causes more than 500,000 infections worldwide (Pappas, Papadimitriou, Akritidis, Christou, & Tsianos, 2006). Brucellosis is more common in areas with poor public health and in countries with the absence or insufficient preventive programs in domestic animals (Capasso, 2002). The disease is widely prevalent and has recently been reported in domestic animals and humans in Oman, Qatar, Kuwait, Saudi Arabia, Iraq, Russia, Turkey, Pakistan and the UAE, which share a vast border with Iran and have close economic relationships (Mohammed & Shigidy, 2013; Wernery, 2014).

Camel brucellosis has been diagnosed in all camel‐rearing countries (Wernery, 2014). Most of the infected camels are asymptomatic carriers of brucellosis. Clinical signs of camel brucellosis are epididymitis and orchitis, lesions of lymph nodes and joint capsules, metritis, abortion and reduced fertility (Esmaeili, 2015; Wernery, 2014).

Human brucellosis is mainly an occupational disease, and the major routes of transmission include contact with animal tissues, blood, urine, vaginal discharge, aborted fetuses, and especially placentas, and consumption of raw milk and other unheated dairy products (Wernery, 2014). Brucellosis is an endemic disease in humans and animals in Iran with an annual incidence of 34 per 100,000 in human (Basiri et al., 2016).

A serological investigation from 1987 to 2014 in various parts of Iran showed that a range of 0.84%–37.83% were positive for *Brucella* (Sazmand, Hajikolayi, Ghorbanpoor, & Hekmaimoghaddam, 2012). In a study in various parts of Iran in 2012, 32.52% of aborted fetus samples from camels were positive for *Brucella* detected by conventional PCR (Dehkordi et al., 2012; Dehkordi and Taghizadeh 2012). In another study on camels from Isfahan, central Iran (2012), 11.42% of the studied aborted fetuses of camels were recognized to be infected by brucellosis through culture and multiplex PCR (Dehkordi et al., 2012; Dehkordi and Taghizadeh 2012).

Camel brucellosis seems to be an important endemic zoonotic disease in Iran. For the eradication of brucellosis in camels and further elimination of the disease in human, it is recommended to carry out the ‘test and slaughter' and ‘vaccination' policy. Screening of camels and controlled movement of infected or suspected animals are recommended to control this infection. The control of camel disease will be more successful if debilitated animals are slaughtered, and other animals such as cattle, sheep and goat are vaccinated.

#### 3.1.4 Campylobacteriosis

*Campylobacter* species are one of the major causes of enteritis in humans and reproductive disease in animals (Salihu, Junaidu, Abubakar, Magaji, & Mohammed, 2009). Campylobacteriosis has been an important infectious disease in both developed and developing countries during the last century (Acheson & Allos 2001).

Studies have indicated that campylobacteriosis is more prevalent in Iran neighbouring countries including Saudi Arabia, Iraq and the UAE (Blaser, 1979; Blaster, Taylor, & Feldman, 1983). Campylobacteriosis can cause abortion, enteritis and lesions in the small and large intestines with typhlocolitis in ruminants, but the clinical presentation in camels has not been described so far (Blaser et al., 1979). The isolation of *Campylobacter* from camels indicates that camel can serve as a source for both man and animal (Salihu et al., 2009). Many animals shed *Campylobacter* spp. in their faeces asymptomatically. Ways of transmission are the consumption of undercooked meat and chicken, environmental exposure and direct contact with such farm animals as camels (Kaakoush, Castaño‐Rodríguez, Mitchell, & Man, 2015).

Campylobacteriosis is an endemic disease in Iran, which was reported in human, sheep, cattle, chicken and camel from different areas, especially central provinces of Iran (Pour Reza, 2013).

A survey in Fars and Bushehr provinces, south of Iran, in 2006 showed 2% infection in camel faeces (Baserisalehi, Bahador, & Kapadnis, 2007). Another investigation during 2007–2008 in Najaf Abad, central Iran, showed 5.3% infection in camel meat (Rahimi, Ameri, & Kazemeini, 2010). A microbiological investigation of the raw camel meat in Isfahan and Yazd provinces, central Iran, in 2008 and 2009 revealed infection in 0.9% of samples (Rahimi et al., 2010). In a survey during 2009 and 2010 in Chaharmahal and Bakhtiari and Khuzestan provinces, southwest of Iran, 2.3% of camel meat was positive for *C. jejuni* and *C. coli* (Rahimi, Ameri, Alimoradi, Chakeri, & Bahrami, 2013).

This evidence suggests that camel and its meat can be a source of *Campylobacter* spp. infection in humans in Iran. Hence, monitoring programs and inspections are necessary to prevent outbreaks of such food‐borne diseases. It is also suggested to study more on this disease to figure out the clinical signs and its importance in transmitting diseases to humans in Iran.

#### 3.1.5 Plague

Human plague is an acute and sometimes lethal bacterial disease caused by *Yersinia pestis* (Butler, 2009). Plague caused three major pandemics during the history all over the world (Mladenova‐Hristova & Tsacheve, 2014). Currently, endemic foci of plague are primarily seen in central and southeast Asia, Africa, and south and northwest of America (Mladenova‐Hristova et al., 2014). Human plague has been reported in camelid and camel playing a very important role in disease transmission to humans (Wernery & Kaaden, 2002). Outbreaks of plague in camels have been reported in Mongolia, China, India, Iran, Iraq, Russia and Africa (Wernery & Kaaden, 2002). Moreover, outbreaks of plague have been reported from Russia through consumption of infected camel milk, and in Afghanistan (2007; Leslie et al., 2011), Saudi Arabia (1994; Saeed, Al‐Hamdan, & Fontaine, 2005; Wernery & Kaaden, 2002), Libya (1977; Misonne, 1977), and Jordan (1997; Arbaji et al., 2005) through consumption of infected camel meat. The disease exists in three clinical forms, namely bubonic, pneumonic and septicemic. The main form of plague in camels is the bubonic form that causes abscesses disseminated over the entire body, pulmonary and cutaneous manifestation, and septicemia (Fedorov, 1960).

Wild rodents are the natural sources of plague. Plague is routinely transmitted to humans and animals by the bite of infected fleas; however, consumption of milk or meat from infected animals can be another rare way of disease transmission in human (Wernery & Kaaden, 2002). Human plague has been reported from all parts of Iran during history, and the country has experienced outbreaks of plague for several centuries (Hashemi Shahraki, Carniel, & Mostafavi, 2016). Besides, there are records of camel plague in Iran in the past (Wernery & Kaaden, 2002). In 1897 and 1907, a few plague‐infected camels were noticed in some areas of Iran (Fedorov, 1960; Mcgrane et al., 1985). In 1974, camel plague transmission was reported in southern areas of the Caspian Sea in the north, and in Kurdistan province (Mesopotamia) in the west of Iran (Fedorov, 1960), the latter is still one of the endemic focus of plague in the world. A serological investigation in an area between Kurdistan and Hamadan in 2011 and 2012 reported that rodents and sheepdogs in this region were positive for plague, emphasizing the area as an active endemic region (Esamaeili et al., 2013).

*Yersinia. pestis* infection is very critical to health in both humans and animals. According to previous reports, human plague is an endemic disease in western Iran, with incidences of camel plague in the neighbouring countries such as Afghanistan, Jordan and Saudi Arabia. Camel plague, therefore, needs to be considered in both native and imported camels in Iran, necessitating a comprehensive study on plague infection in samples of native and imported camels.

#### 3.1.6 Tuberculosis

Tuberculosis (TB) is one of the major zoonotic diseases in animals and humans caused by the bacterial genus *Mycobacterium* (Thoen, 2014). Tuberculosis caused millions of human death worldwide when there was no adequate treatment in the past (Ducati, Ruffino‐Netto, Basso, & Santos, 2006). *Mycobacterium bovis* is an important zoonotic agent (Thoen, LoBue, & De Kantor, 2006). Outbreaks of bovine tuberculosis are a concern for public health authorities (Wernery & Kinne, 2012). There are several reports published on camel tuberculosis in Egypt, Somalia, Ethiopia, UAE, Pakistan, Australia, Dubai, India, Germany, USA, Mauritania and Russia (Mamo et al., 2011; Wernery, 2014; Wernery & Kinne, 2012). Clinical signs of *M. bovis* in camelids include wasting, anorexia, respiratory distress, enlargement of superficial lymph nodes, recumbency and eventually death (Wernery & Kinne, 2012). Tuberculosis can spread to human through ingestion of raw milk, and sometimes by inhalation of infectious droplets (Wernery & Kinne, 2012).

Tuberculosis exists in all provinces in Iran with the highest incidence and prevalence in Sistan and Baluchestan and Golestan provinces (Metanat, Sharifi‐Mood, Alavi‐Naini, & Aminianfar, 2012). In 2015, a proportion of 12.59 per 100,000 Iranians suffered from tuberculosis. In 2002, a study on camel meats from slaughterhouses in Mashhad, northeastern Iran, showed that tuberculosis was prevalent in camels of the region (Hashemi, Torabi, Darabi, & Shirazi, 2002). An investigation on mediastinal, bronchial and retropharyngeal lymph nodes from 102 dromedary camels slaughtered in Mashhad Abattoir from 2009 to 2010 showed 16.66% infection among samples (Soleymani Babadi et al., 2012).

A program to control tuberculosis in camelids based only on intradermal tuberculin tests will face severe deficiencies. Other than the intradermal test, antemortem tests, such as lymphocyte transformation and ELISA tests, have also failed to be adequately reliable in undomesticated mammals because of false‐negative and false‐positive reactions. This is also true for tuberculosis testing in camelids. However, it is recommended to use several tests to aid in diagnosing tuberculosis in camelids (Fowler, 1999). Using improved strategies to screen camel is suggested to figure out the epidemiology, clinical signs and the importance of this animal in transmitting TB to humans in Iran and all over the world.

#### 3.1.7 Pasteurellosis

Pasteurellosis is a zoonotic disease with a tendency for opportunistic infection (Yasutomo & Kazunari, 2005). *Mannheimia haemolytica* and *Pasteurella multocida* are well established to be the major aetiological agents of many pasteurellosis outbreaks (Mohamed & Abdelsalam, 2008). Both species are commensal residents in the upper respiratory tract of healthy cattle, camel, sheep, dog, cat, horse, etc. (Mohamed & Abdelsalam, 2008). Carriage rates of the bacteria are quite high in the oral or nasal secretions of animals. Infection in human is a worldwide problem resulting from animal bites or contact with nasal secretions (Mohamed & Abdelsalam, 2008).

The worst outbreaks in camels occur during the rainy season as the animals are in poor physical conditions (transportation over long distances, deficiencies of vitamins and minerals, and heavy parasitic infestations). Pasteurellosis in camels is reported in Algeria, Egypt, India, Libya, Mauritania, North Africa, Somaliland, Soviet Union, Sudan and Chad (Mustafa, 1987). *Pasteurella* in camels shows a range of pulmonary and septicemic diseases (Momin, Pethkar, Jaiswal, & Jhala, 1987). In Iran, clinical outbreaks of camel pasteurellosis were reported in 1936, 1943 and 1969 (Wernery & Kaaden, 2002). In 2009, 53% of camels got infected, and 10 of them were lost in an outbreak in Larestan, Fars province, south of Iran (Esmaeili et al., 2010). From 2012 to 2013, PCR assay in camels of Tehran abattoir indicated that *P. multocida* prevalence ranged from 7.1% to 14.5% in lung samples (Chitgar, Haghdost, Jamshidian, & Hesaraki, 2014). In a study in 2014 and 2015 in Larestan, Fars provinces, south of Iran, 80% of dead camels, 64.58% of sick camels and 7.34% of samples from healthy camels were positive for *P. multocida (*Tahamtan, Amrabadi, Shahriari, & Namavari, 2017 *).*

Further studies are needed to clarify the epidemiology and risk factors of camel pasteurellosis in Iran. Vaccination is highly recommended for control of disease in Iran.

#### 3.1.8 Salmonellosis

*Salmonella* is one of the leading causes of food‐borne gastroenteritis. Over 2,500 identified serovars of *Salmonella* spp. are responsible for infections in humans and animals globally (Mohamed & Suelam, 2010). Around 40,000 cases of salmonellosis are reported annually. Animals are the principal source of this pathogen. *Salmonella* spp. are mainly transmitted via the faecal–oral route (Chiu, Su, & Chu, 2004). Foods from animal sources such as beef, poultry meat, egg and milk are the most common sources of human salmonellosis (Oosterom, 1991; Salehi, Mahzounieh, & Saeedzadeh, 2005).

*Salmonella* infection in camels is reported from Sudan, Palestine, France, North Africa, USA, Somalia, Ethiopia, Egypt, UAE and Iran. In camels, *Salmonella* disease can cause enteritis, septicemia and abortion (Sepehr, 2012). Chronic salmonellosis in camels is characterized by diarrhoea, weight loss and death within a few weeks (Wernery & Kaaden, 2002). Healthy camels can be the carriers of *Salmonella* spp. (Salehi et al., 2005). Humans can be infected by the consumption of contaminated foods originated from camels, infected drinking water, or close contact with infected camels (Wernery & Kaaden, 2002).

Serological investigations showed that *Salmonella* infection in camel faeces samples varied between 4% in 1992 and 12.39% in 2013 (Moghaddas, 2012 2012)*.* Similar studies on slaughtered camels indicated that 37.25% and 1.69% of camel samples were infected by *S. Typhi* in the north and centre of Iran in 1992 and 1994, respectively (Miranzade, 1994). A microbiological investigation on tissue samples of camels in Tehran, north of Iran, in 2010 showed that 1.1% were contaminated with *S. Typhimurium* (Nour‐Mohammadzadeh et al., 2010). Cross‐sectional studies on camels revealed that 43% and 100% of camel milk samples were infected by *Salmonella* spp. in rural areas and Golestan province, Iran, during 2012 and 2015, respectively (Sepehr, 2012).

In 2012, 10% of camel meat samples from Mashhad, northeast of Iran, were reportedly infected by S*almonella* spp. (Golami & Seyedin, 2011). In 2016, an outbreak of abortion and diarrhoea was reported in a camel herd in Mashhad, northeast of Iran. The presence of *Salmonella* in straw and beet pulp sampled from rumen contents of camels was recognized in two of ten cases by microbiological tests (Mohammady & Najafi Mosleh, 2017).

According to the studies in Iran, camels are an important source of *Salmonella*. It is, therefore, important to control and prevent salmonellosis in these animals and their products to decrease the transmission of this agent to a human.

#### 3.1.9 Escherichia coli

*E. coli* O157: H7 is food and water‐borne zoonotic agent (García, Fox, & Besser, 2010). Infection caused by this bacterium is usually asymptomatic in domestic animals and wildlife (Rahimi, Kazemeini, & Salajegheh, 2012; Suardana, Widiasih, Mahardika, Pinatih, & Daryono, 2015). Human infection usually occurs due to improper hygiene, ingestion of contaminated food and water, or direct contact with infected animals (Ateba & Mbewe, 2014; Bogard, Fuller, Radke, Selman, & Smith, 2013). Human *E. coli* O157: H7 infections have been reported in more than 30 countries (Rahimi et al., 2012). *E. coli* enterotoxaemia is reported in dromedary camels, and sporadic cases have been reported in adult breeding camels in the UAE, Bahrain, Sudan and East Africa (Al‐Ruwaili, Khalil, & Selim, 2012; Wernery & Kaaden, 2002). Affected animals develop a disease with evidence of yellowish watery diarrhoea, sunken eyes, abdominal cavity distention and CNS signs in some dromedaries (Al‐Ruwaili et al., 2012). Sporadic cases of *E. coli* infection were reported from several parts of Iran (Koochakzadeh, Badouei, Mazandarani, & Madadgar, 2014), but there is limited information regarding the prevalence of *E. coli* O157: H7 in Iranian camels (Rahimi et al., 2012). In a study on camel meat samples from Isfahan, Shahrekord, Yazd, Fars and Khuzestan from 2006 to 2010, 1.1% to 2% of samples were infected by *E. coli* O157: H7 (Rahimi et al., 2010).

Due to rare positive reports provided on camels in Iran, it seems that camel meat is not an important source for *E. coli* O157: H7 infection; however, monitoring and inspection programs on camel meat and its products remain an important strategy to prevent outbreaks of such a food‐borne disease.

### 3.2 Listeriosis

Listeriosis is one of the major zoonotic food‐borne diseases (Al‐Swailem et al., 2010). Cattle, sheep, camels and humans are the sources of this globally distributed disease (Heymann, 2015) being more common in regions with cold temperatures. The annual incidence of listeriosis in humans varies from 0.1 to 11.3 cases per 1,000,000 in different countries (Swaminathan & Gerner‐Smidt, 2007).

*Listeria* contains seven species, of which *L. monocytogenes* infects both humans and animals (Rahimi, Momtaz, Behzadnia, & Baghbadorani, 2014). *Listeria monocytogenes* is very resistant to dryness and may stay viable in dry soils and faeces for up to 2 years. This microorganism could be found in the soil, vegetables, sewage, genital secretions and nasal mucous membrane of healthy animals (Al‐Swailem et al., 2010; Dehkordi, Barati, Momtaz, Ahari, & Dehkordi, 2013; Dehkordi, Haghighi Borujeni, Rahimi & Abdizadeh, 2013). Listeriosis infects camels due to consuming spoiled forage infected with *L. monocytogenes* (Safdari & Jahantigh, 2014). Listeriosis causes a disease with signs of meningoencephalitis in new word camels that include circling, trembling of the head, running into objects and fever. Some cases develop unilateral facial nerve paralysis in association with drooping lips, ears, eyelids and paralysis of the jaw and pharynx, which interfere with mastication and swallowing (Wernery & Kaaden, 2002). Human listeriosis is associated with consumption of contaminated milk, soft cheese and undercooked meat from infected animals (Rahimi et al., 2014). Reports of human listeriosis in Iran are uncertain (Lotfollahi et al., 2011).

In 2011, a report from Sistan and Baluchistan province showed that 8.75% of camel raw meat samples were infected by *Listeria* bacteria, three samples of which were associated with *L. monocytogenes* and four samples were contaminated with *L. unique* (Safdari & Jahantigh, 2014).

Camel meat samples showed evidence of *Listeria* spp. infections in 50% and 9.6% of cases from Tehran (2011) and Isfahan (2007), respectively (Mashak, Zabihi, Sodagari, Noori, & Akhondzadeh Basti, 2015; Rahimi et al., 2010). Another study conducted in various parts of Iran (2011) detected *L. monocytogenes* infections in milk, urine, faeces and vaginal secretions of camels in 5.94% of camel raw milk, with the highest (15.18%) shedding of *L. monocytogenes* in camel vaginal secretion (Dehkordi, Barati, et al., 2013; Dehkordi, Haghighi Borujeni, et al., 2013).

The results of studies in Iran show that camel meat is an important source of *Listeria* infection for humans; thus, continuous monitoring and inspection programs are necessary to prevent outbreaks of listeriosis in humans.

### 3.3 Viral zoonotic diseases

In this study, rabies, camelpox, MERS‐CoV infection, and CCHF are reported as zoonotic viral infections among camels in Iran (Table 2).

**Table 2:** Summary of zoonotic viral diseases of camels reported in Iran; 1890–2018

B. Viral diseases					
Disease	Year	Location	Techniques	No. positive/ No. tested (%)	Ref
Rabies	1996–2006	Golestan	Histopathological examination, Fluorescent antibody technique	3 cases	(Bokaei et al., 2009)
	2012	Semnan (Torod region)	Histopathological examination, Fluorescent antibody technique	8 cases	(Esmaeili et al., 2012)
	2012	East Azerbaijan (Khoda Afarin)	Histopathological examination, Fluorescent antibody technique	–	(Nadalian, 2012)
Camlepox	1957	Sistan and Baluchestan (Bazman)	–	1 case	(Moghaddas, 2012 2012)
	1979	Sistan and Baluchestan (Bampur and Zabol)	Electron microscopy	–	(Moghaddas, 2012 2012)
	1984	Isfahan, Kerman, Fars, Khuzestan, Semnan, Zabol, Bampur	Electron microscopy	Outbreak	(Moghaddas, 2012 2012)
	1993	Kerman (Zarand)	Electron microscopy	50/80 (62.5%)	(Rashidi, 1993)
	1996	Sistan and Baluchestan (Iranshahr(Delgan))	Electron microscopy	3/50 (6%)	(Moghaddas, 1998)
	2000	Tehran(Pishva)	Electron microscopy	2/110 (1.81%)	(Moghaddas, 2012 2012)
	2014	Qum, South Khorasan, Sistan and Baluchestan (Khash)	PCR (Bioneer Kit)	(100%) No Numeral data	(Mosadeghhesari et al., 2014)
MERS‐CoV	2014	Sistan and Baluchestan (Zabol city)	Serology test	3/18 (16.66%)	(Khalaj, 2014a)
	2014	Sistan and Baluchestan (Zabol and Mirjaveh)	Serology test	8/186 (4.30%)	(Khalaj, 2014b)
	2014	Kerman and West Azerbaijan	RT‐PCR	7/98 (7.14%)	(Khalili Bagaloy et al., 2017)
CHHF	2014	North Khorasan, South Khorasan, Razavi Khorasan	ELISA	9/136 (5.29%)	(Champour et al., 2014)

#### 3.3.1 Rabies

Rabies is a severe and widespread zoonotic disease (Blancou, 1988) caused by a group of neurotropic viruses from the genus *Lyssavirus* of the Rhabdoviridae family, sometimes known as genotype 1 virus to distinguish it from other closely related viruses causing similar illnesses (Hyun et al., 2005; Sacramento, Badrane, Bourhy, & Tordo, 1992). Rabies virus has been isolated from nearly all mammalians. Herbivores and man are the final hosts and fail to normally play a role as vectors. Carnivorous and vampire bats are considered the sources of the virus (Nigg & Walker, 2009; Rupprecht, Hanlon, & Hemachudha, 2002). More than 55 000 people die of rabies annually mostly in Asia and Africa(Chaurasia, 2014). Rabies has been reported in camels from Morocco, Mauritania, Sudan, Yemen, Saudi Arabia, UAE, Niger, Jordan, India, Israel and Iran (Abbas & Omer, 2005; Fassi‐Fehri, 1987). Rabies in dromedary camel occurs in two forms of ‘raging fury’ and ‘silent fury’, the latter, however, is rarely seen in camels. Raging fury includes two of excitative (furious) and paralytic phases (Wernery & Kaaden, 2002). Infected animals transfer the virus to other animals and humans via saliva following a bite or scratch (Gholami, Fayaz, & Farahtaj, 2014).

Iran is highly endemic for rabies, where it can easily circulate in wildlife and livestock (Farahtaj, Fayaz, Howaizi, Biglari, & Gholami, 2014; Janani et al., 2007). This disease is widespread in all provinces, especially in the north, northwest and northeast regions of the country (Simani, Gholami, Farahtaj, Yousefi‐Behzadi, & Fayaz, 2001), occasionally reported in camelids of Iran (Esmaeili, Ghasemi, & Ebrahimzadeh, 2012). Most of the rabid camels were reported from Sistan and Baluchistan region, southeastern Iran (Simani, 2003; Simani et al., 2001).

Three rabid camels were also reported from Golestan province during 1996–2006 Bokaei, Fayaz, Pourmehdi, and Haghdoost (2009). In 2008, an outbreak of camel rabies was reported for the first time in Torod region of Semnan province, central Iran. Eight infected camels were attacked by a rabid wolf. Another camel rabies was reported from Khoda Afarin county in East Azerbaijan province, northwest of Iran, in 2012 (Moghaddas, 2012 2012).

There is a need for more studies on camels in Iran to have a better overview of the rabies situation in these animals. As this virus is neurotropic and highly fragile, the last line of more studies may be deleted, except those reporting the disease in camel/other animals. Further investigations may include rather prompt vaccination of suspected exposed animals and prevention from exposure to rabid and wild animals.

#### 3.3.2 Camelpox

Camelpox is an important contagious skin disease of camelids (Balamurugan, Venkatesan, Bhanuprakash, & Singh, 2013). The causative agent of the disease is the camelpox virus (CMLV) belonging to the family Poxviridae. This disease can be pathogenic for human as well (Prabhu et al., 2015). Camelpox is mostly reported from Asia (Iran, Iraq, Saudi Arabia, UAE, Yemen, Syria, India, Afghanistan and Pakistan), Africa (Algeria, Egypt, Kenya, Mauretania, Niger, Somalia, Morocco, Ethiopia, Oman and Sudan) and the southern parts of former USSR (Wernery, Kaaden, & Ali, 1997). The clinical manifestation of camelpox varies from mild local to severe systemic disease. The disease is characterized by an initial rise in temperature, followed by enlarged lymph nodes, skin lesions (erythematous macules, papules, vesicles and pustules followed by crusts from ruptured pustules), and prostration (Balamurugan et al., 2013; Wernery & Kaaden, 2002; Wernery et al., 1997). Infected camels shed the virus into secretions including saliva, milk, ocular discharge, nasal discharges and dried scab. The route of transmission is via inhalation, skin abrasions and tick bite (Prabhu et al., 2015). The first official report of camelpox was from one camel in Bazman region of Sistan and Baluchistan province in 1957 (Moghaddas, 2012 2012). Camelpox was also reported from Bampur and Zabol in Sistan and Baluchestan province, southeastern Iran, in 1979 (Moghaddas, 2012 2012). Outbreaks of camelpox were reported in camels from Isfahan, Kerman, Fars, Khuzestan, Semnan, Zabol and Bampur regions in 1984 (Moghaddas, 2012 2012). In 1993, 62.5% of camelpox cases were reported among susceptible camels in Zarand city, Kerman province, south of Iran (Rashidi, 1993). In 1996 and 2000, 6% and 0.018% of camels were infected with camelpox in Sistan and Baluchistan province and Tehran province (Pishva), respectively (Moghaddas, 2012 2012). In an outbreak of camelpox in 2014, scabs from skin lesions were collected from infected camels from Qom province (Khash city) in central Iran, and Sistan and Baluchistan province in southeastern Iran, and South Khorasan province in eastern Iran. Samples were nearly 100% identical to each other and to camelpox strains CMS and M‐96. These isolates also had 99% and 95% similarity to CP‐1 strain and FIN/T2000 isolate, respectively (Mosadeghhesari, Oryan, Zibaee, & Varshovi, 2014).

The vaccination program has been developed to prevent and control camelpox in almost any camel raising country and some other countries (e.g., Saudi Arabia, UAE and Morocco) that have camel racing competitions (Higgins, 1992). Besides, the live‐attenuated cell culture camelpox vaccine is currently used in many countries, such as Afghanistan, Bahrain, Iraq, Jordan, Kuwait, Lebanon, Oman, Pakistan, Syria, UAE, Yemen, Egypt, Morocco and Russia (Bhanuprakash et al., 2010), various inactivated camelpox vaccines were used in Saudi Arabia (Khalafalla & El‐Dirdiri, 2003) and UAE (Wernery & Kaaden, 2002). No inactive or live vaccine against camelpox has yet been produced in Iran. Because of the importance of camelpox and the lack of a comprehensive study on this disease in Iran, more monitoring programs and inspections are necessary to prevent outbreaks of this disease and its economic loss.

#### 3.3.3 MERS‐CoV

MERS‐CoV is a novel coronavirus that causes severe acute respiratory disease. First reported in Saudi Arabia in 2012 (Gonzalez Gompf, 2015), the disease has been described primarily in countries of the Middle East including Saudi Arabia, Qatar, UAE, Jordan, Kuwait, Lebanon, Oman, Iran and Yemen. Imported cases have also been reported in Algeria, Austria, China, Egypt, France, Germany, Greece, Italy, Malaysia, Netherlands, Philippines, South Korea, Thailand, Tunisia, Turkey, UK and USA (Ramadan & Shaib, 2019). MERS‐CoV has been found in many dromedary camels (Mackay & Arden, 2015) and is enzootic in camels across the Arabian Peninsula and parts of Africa, causing mild upper respiratory tract disease in its camel host. MERS‐CoV infects dromedary camels and can be transmitted to human through close contact. Camels are infected transiently, and it seems that the virus will be cleared after the acute infection. Camels may also act as intermediate hosts by transmitting the virus from its origin to humans. The exact source that maintains the virus has not yet been identified (Azha et al., 2014).

In October 2014, three cases of MERS‐CoV‐related coronaviruses were reported among 18 susceptible camels in Zabol city, Sistan and Baluchistan province, southeastern Iran. The source of infection was due to the animals imported illegally from Pakistan (Khalaj, 2014a). In December 2014, eight illegally imported camels from Pakistan to Iran were infected with a coronavirus ‘potentially related’ to MERS‐CoV. The animals were among 186 camels that were tested at two border stations, namely, Zabol and Mirjaveh, in Sistan and Baluchistan province (Khalaj, 2014b).

Six cases of MERS‐CoV infection were reported from Iran, five cases during May–July 2014 and one case in March 2015 in Kerman province, southern Iran. The cases presented epidemiological data related to those who had returned from Hajj (Moniri, Marjani, Tabarsi, Yadegarynia, & Nadji, 2015; Mortazavi, Monavari, Ataei Pirkooh, & Tavakoli, 2014).

In 2014, a molecular study showed pancoronavirus RNA in 7.14% of nasal swab samples from apparently healthy camels. Positive samples (4.08%) belonged to West Azerbaijan province, northwest of Iran, and the remaining were obtained from Kerman province, southeast of Iran (Khalili Bagaloy, Sakhaee, & Khalili, 2017). Following surveillance and monitoring across the country, there have been no reports of further animal and human cases of MERS‐CoV in Iran. Camels imported from the neighbouring countries should be strictly monitored continuously.

#### 3.3.4 Crimean‐Congo haemorrhagic fever

CCHF is a tick‐borne viral zoonotic disease caused by one of the most medically important arboviruses belonging to the genus *Orthonairovirus* in the family Nairoviridae (Fajs et al., 2014; Shayan, Bokaean, Shahrivar, & Chinikar, 2015). The most common viral sources are domestic livestock including sheep, goat, cattle and camel, which show asymptomatic infections (Schwarz et al., 1996). The virus transmission to humans has been reported through an infected tick bite, direct contact with fresh meat or blood of viraemic animals and nosocomial infection (Schwarz et al., 1996; Shayan et al., 2015).

CCHF is one of the most widespread and common tick‐borne viral diseases (Aslam et al., 2016). The geographical distribution of the disease overlaps with that of *Hyalomma* ticks (Soares‐Weiser, Thomas, G, & Garner, 2010). CCHF is endemic in some parts of Africa, the Middle East and southeastern Europe (Fajs et al., 2014). Positive serological tests have been reported in Oman (Body et al., 2016), Iran and Niger (Mariner, Morrill, & Ksiazek, 1995) among tested camels.

The disease has been reported from most parts of Iran. Most patients are reported from Sistan and Baluchistan province, southeast of the country, due to a long border with two high‐risk countries, Afghanistan and Pakistan (Chinikar et al., 2012; Telmadarraiy, Chinikar, Vatandoost, Faghihi, & Hosseini‐Chegeni, 2015). CCHF antibodies are found in Iranian sheep (38%), goats (36%) and cattle (18%) (Saidi, Casals, & Faghih, 1975). In 2014, a seroepidemiological study of CCHF virus in camels of Khorasan provinces (North, South and Razavi), northeast of Iran, showed that from 170 camels collected from different regions of Khorasan territory, a total of 5.29% camels were IgG‐positive. Eight of the nine positive samples were taken from female camels (Champour et al., 2014).

As CCHF is a serious infectious disease, imported animals, particularly camels that carry a large number of ticks should be screened more carefully at border points of the country or, alternatively, camels should be slaughtered at border cities allowing the import of processed meat.

### 3.4 Parasitic zoonotic diseases

Echinococcosis, cryptosporidiosis and toxoplasmosis have been reported as zoonotic parasitic diseases in Iranian camels (Table 3).

**Table 3:** Summary of zoonotic parasite diseases of camels reported in Iran; 1890–2018

C. Parasite infections					
Disease	Year	Location	Techniques	No. positive/No. tested (%)	Ref
Echinococcosis	1970	Tehran	Direct light microscopic examination	612/955 (64%)	(Mobedi et al., 1970)
	1971	Shiraz	Direct light microscopic examination	15/35 (42.8%)	(Afshar et al., 1971)
	1992	Shiraz	Direct light microscopic examination	28/40 (70%)	(Moghadar et al., 1992)
	1994	Isfahan, Yazd	Direct light microscopic examination	70/100 (70%)	(Mowlavi, 1997)
	2001	Yazd, Isfahan, Kerman	Direct light microscopic examination	37/144 (25.7%)	(Anvari et al., 2001)
	2001–2002	Yazd, Kerman, Zahedan, Mashhad, Isfahan	Direct light microscopic examination	26.5%, 25.7%, 32.7%, 59.3%, 35.2%, Total: 233/661 (35.5%)	(Ahmadi, 2005)
	2002	Tehran, Isfahan, Yazd	Molecular, PCR, RFLP	23 Cases	(Ahmadi & Dalimi, 2002)
	2006–2008	Golestan	Direct light microscopic examination	403/1341 (30.1%)	(Mostafalu & Rahchamani, 2016)
	2009	Kerman	Direct light microscopic examination	45/217 (20.73%)	(Fathi, Mirzaei Dehaghi, & Radfar, 2012)
	2009	Razavi Khorasan (Mashhad)	Direct light microscopic examination	85/207 (41.1%)	(Sharifiyazdi, Oryan, Ahmadnia, & Valinezhad, 2011)
	2009–2010	Yazd	Serological, IH, CIEP	18/141 (12.85%) IH, 16/141 (11.3%) CIEP	(Sazmand et al., 2013)
	2010–2011	Khorasan Razavi, Yazd, South Khorasan, Semnan, Sistan va Baluchestan	Direct light microscopic examination	54/136 (40.40%), 25/80 (31.20%), 32/120 (26.60%) 6/27 (22.20%), 18/75 (24.00%), Total: 135/438 (30.82%)	(Moghaddas, Borji, Naghibi Aboul, Razmi, & Shayan, 2014)
	2016	South Khorasan Sistan va Baluchestan	Direct light microscopic examination	85/207 (41%)	(Valinezhad & Oryan 2017)
	2015–2016	Semnan	Direct light microscopic examination	221/475 (46/52%)	(Darebaghi Hosein Abadi, Najmoddin, Keneshlou, & Kordi, 2016)
Cryptosporidium spp	2009	Isfahan(Najaf Abad)	Microscopic examination of smears stained by modified Ziehl‐Neelsen technique	39/103 (37.9%)	(Razavi et al., 2009)
	2009	Razavi Khorasan (Mashhad)	Microscopic examination of smears stained by modified Ziehl‐Neelsen technique	6/316 (1.9%)	(Borji et al., 2009)
	2010	Yazd	Microscopic examination of smears stained by modified Ziehl‐Neelsen technique	61/300 (20.33%)	(Sazmand, Rasooli, et al., 2012)
	2010	Qeshm Island southern Iran	Microscopic examination of smears stained by modified Ziehl‐Neelsen technique	11/65 (16.9%)	(Nazifi et al., 2010)
	2012	West Azerbaijan (Miandoab region)	Microscopic examination of smears stained by modified Ziehl‐Neelsen technique	17/170 (10%)	(Radfar, Gowhari, & Khalili, 2013)
	2012	Kerman (Shahr‐e Babak)	Microscopic examination of smears stained by modified Ziehl‐Neelsen technique, ELISA	4/85 (4.7%)	(Radfar et al., 2013)
	2016	Razavi Khorasan	Microscopic examination of smears stained by modified Ziehl‐Neelsen technique, PCR	4/60 (6.6%)	(Sadrbazzaz et al., 2016)
Toxoplasmosis	2006	Razavi Khorasan (Mashhad)	Serological screening indirect fluorescent antibody test (IFAT)	5/120 (4.16%)	(Sadrebazzaz et al., 2006)
	2011–2012	Tehran, Isfahan, Fars	Cell line culture, enzyme‐linked immunosorbent assay (ELISA), PCR	5/160 (3.12%)	(Dehkordi, Barati, et al., 2013; Dehkordi, Haghighi Borujeni, et al., 2013)
	2013	Isfahan	PCR	8/122 (6.6%) 0/36 (0%)	(Khamesipour, Doosti, et al., 2014; Khamesipour, Rahimi, et al., 2014)
	2014–2015	Razavi Khorasan (Sabzevar)	PCR	26/40 (65%)	(Aliabadi & Ziaali, 2016)

#### 3.4.1 Echinococcosis

Human echinococcosis is a zoonotic infection caused by larval forms (metacestodes) of tapeworms of the genus *Echinococcus* (Grosso, Gruttadauria, Biondi, Marventano, & Mistretta, 2012; Otero‐Abad & Torgerson, 2013). The most important genera for public health are *E. granulosus* and *E. multilocularis* (Grosso et al., 2012). Present estimates suggest that it results in the loss of 1–3 million disability‐adjusted life per year (El Berbri et al., 2015). Typical hosts are dogs and other carnivores, and intermediate hosts are ungulates such as sheep, goats, horses and camels. Echinococcosis in camel usually causes no significant clinical signs, but, there are occasional reports concerning respiratory problems, abdominal distention and death from anaphylaxis (Wernery & Kaaden, 2002). Humans as the accidental hosts are infected by oral ingestion of eggs that contaminated vegetables, water, foods or hands (Eckert & Deplazes, 2004).

The disease is endemic to hyperendemic in agricultural countries of Europe, Africa, America, the Middle East and Asia (Moro & Schantz, 2009). Camel echinococcosis is reported from North and East Africa, Egypt, Sudan, Somalia, Libya, Central Africa, Iraq and Iran (Wernery & Kaaden, 2002). There are few data about the prevalence of echinococcosis in dromedaries from the Middle East countries including Iran.

There are many reports of human echinococcosis cases as a health problem distributed throughout Iran. It is estimated that echinococcosis is responsible for almost 1% of surgical operations in Iran (Rokni, 2009). Information on the epidemiology of echinococcosis in camels in different regions of Iran is very limited (Borji & Parandeh 2010). Cross‐sectional studies and parasitology tests from 1970 to 2016 showed that infected camels with *E. granulosus* varied from 20.73% to 70% (Afshar, Nazarian, & Baghban‐Baseer, 1971; Mobedi, Madadi, & Arfaa, 1970; Moghadar, Oryan, & Pour, 1992; Mowlavi, 1997; Sazmand & Joachim, 2017) in various parts of Iran (Table 3). A seroepidemiological study from 2009 to 2010 in Yazd province, central Iran, disclosed that 12.8% of camels were positive with indirect haemagglutination (Sazmand, Razi Jalali, Hekmatimoghaddam, & Asadollahi, 2013).

As it can be observed from different studies in Iran, at least 12% of the camels in all parts of the country are infected with echinococcosis, which is a high rate of infection. Thus, camels can play an important role in the transmission of *Echinococcus* in Iran, and the camel carnivorous cycle should also be targeted in any control program.

#### 3.4.2 Cryptosporidiosis

Cryptosporidiosis is one of the intracellular protozoal diseases caused by the genus *Cryptosporidium* (Rossle & Latif, 2013) infecting fish, amphibians, reptiles, birds and mammals. It has been identified as the cause of numerous outbreaks of diarrhoeal disease in humans and animals all over the world (Fayer, 2004; Razavi, Oryan, Bahrami, Mohammadalipour, & Gowhari, 2009). *Cryptosporidium* is transmitted via the faecal–oral route and easily spreads through water, food and contact with infected animals and contaminated environments (Lal, Cornish, Fearnley, Glass, & Kirk, 2015; Sazmand & Joachim, 2017). Cryptosporidiosis is a zoonotic problem and its excreted oocysts could be the sources of human infection and great public health concern (Pieniazek et al., 2003).

*Cryptosporidium* sp. is reported in dromedary camels in Egypt (Abou‐Eisha, 1994) and Iraq (Hussin, Khalaf, & Ali, 2015). The disease in camel can lead to severe diarrhoea, emaciation, dehydration and death (Wernery & Kaaden, 2002). In Iran, epidemiological studies reported a wide range of infections from 0% to 32% in different human societies (Jafari, Maghsood, & Fallah, 2012). Few data are available on *Cryptosporidium* prevalence in camels of Iran (Nazifi et al., 2010; Sazmand & Joachim, 2017). From 2008 to 2012, microscopic examination of smears stained by modified Ziehl‐Neelsen showed that 1.9% to 37.9% of tested camels (*Camelus dromedarius*) were positive for *Cryptosporidium* oocysts (Borji, Razmi, Movassaghi, Naghibi, & Maleki, 2009; Razavi et al., 2009) in various parts of Iran (Table 3). In 2009 and 2010, a study in Yazd province, central Iran, presented evidence that the rates of infection in faeces and abomasal mucosa of camels were 20.33% and 12%, respectively (Sazmand, Rasooli, Nouri, Hamidinejat, & Hekmatimoghaddam, 2012). In the last reviewed study in Khorasan Razavi province (2016), 6.6% of camel faeces were infected with *Cryptosporidium* spp., and three of the four samples were *C. andersonii*, and one of the four samples was *C. parvum* (Sadrbazzaz et al., 2016).

According to different studies on camels in Iran, camels can be a potential source of cryptosporidiosis infection for human; therefore, slaughterhouse staff, farmers and veterinarians, who have close contact with camels, are at risk of infection and should wash their hands carefully to reduce the risk of infection.

#### 3.4.3 Toxoplasmosis

Toxoplasmosis is a cyst‐forming parasitic disease caused by *Toxoplasma gondii* (Tenter, Heckeroth, & Weiss, 2000). It is an intestinal coccidial parasite of Felidae, particularly cats, which is a primary host. The parasite also infects humans, ruminants and other mammalians as intermediate hosts (Dubey, 2009). Infection with *T. gondii* has a worldwide distribution, and at least one‐third of the world population is infected with the parasite (Robert‐Gangneux & Darde, 2012). Toxoplasmosis is considered a food‐borne infection observed in many domesticated or food animals (Sarvi et al., 2015). The overall seroprevalence rate of toxoplasmosis among the general population is 39.3% in Iran. *Toxoplasma* antibodies in camels are reported in Egypt, Nigeria, Saudi Arabia, Sudan, Turkmenistan, UAE, India and Iran (Hussein Khalil, 2015). Toxoplasmosis in camel causes mild disease with dyspnoea associated with pyothorax and abortion (Wernery & Kaaden, 2002). Human infection may result from ingestion of cysts with bradyzoites, oocysts from cat faeces (e.g. on vegetables) and trans‐placental spread of tachyzoites to the fetus during acute infection in pregnancy. Camels acquire the infection by ingesting food contaminated with oocysts. Cats given camel meat excreted oocysts of *Cystoisospora felis*, *C. rivoltu* and *T. gondii* (Hilali, Fatani, & Al‐Atiya, 1995). Humans acquire the infection from camels by eating raw or undercooked camel meat contaminated with tissue cysts (Hilali & Fahmy, 1993; Wernery & Kaaden, 2002).

In 2004–2005, antibodies against *T. gondii* were found in 2.5% of 120 camels tested in Mashhad (Sadrebazzaz, Haddadzadeh, & Shayan, 2006). In 2011–2012, *T. gondii* was shown to be present in 3.12% of camel milk samples in Tehran, Isfahan and Fars provinces (Dehkordi, Barati, et al., 2013; Dehkordi, Haghighi Borujeni, et al., 2013). In 2013 and 2015, studies on *T. gondii* in camel blood samples showed that 6.6% and 65% of camels were infected in Isfahan and Khorasan Razavi provinces, respectively (Khamesipour Doosti, Mobarakeh, & Komba, 2014; Khamesipour, Rahimi, Shakerian, Doosti, & Momtaz, 2014; Sazmand & Joachim, 2017). Consumption of undercooked camel meat may constitute a risk of infection to humans and should, therefore, be of public concern. The camel–cat cycle should also be targeted in any control program of toxoplasmosis in Iran.

### 3.5 Mycotic zoonotic diseases

There is only one fungal zoonotic disease, viz., dermatophytosis (ringworm), reported for camels (Table 4).

**Table 4:** Summary of zoonotic fungal diseases of camels reported in Iran; 1890–2018

D. Fungal diseases					
Disease	Year	Location	Techniques	No. positive/No. tested (%)	Ref
Ringworm	1994–1998	Different areas of Iran	Direct microscopic examination	14/63 (22.22%)	(Khosravi & Mahmoudi, 2003)
	2003–2013	Different area of Iran	Direct microscopic examination	74/100 (74%)	(Shokri & Khosravi, 2016)

#### 3.5.1 Dermatophytosis

Dermatophytosis is a zoonotic disease caused by a group of fungi named dermatophytes that can infect the skin, hair and nails (Almuzaini, Osman, & Saeed, 2016; Hayette & Sacheli, 2015). The major sources of the fungi are pets, farm and wild animals (Sharma, Kumawat, Sharma, Seth, & Chandra, 2015a; 2015b). The prevalence of dermatophytes varies in different parts of the world (Sharma, Kumawat, Sharma, Seth, & Chandra, 2015a; Sharma et al., 2015b; Vena et al., 2012).

Dermatophytosis is a common disease of camels worldwide (Almuzaini et al., 2016; Tuteja, Patil, Narnaware, Nagarajan, & Dahiya, 2013a; 2013b) more commonly seen in camels under 3 years of age with a peak incidence age between 3 and 12 months (Fadlelmula, Agab, Le Horgne, Abbas, & Abdalla, 1993). Direct and indirect contacts with infected animals and fomites are the modes of infection transmission to human and other animals and vice versa (Tuteja, Patil, Narnaware, Nagarajan, & Dahiya, 2013a; Tuteja et al., 2013b; Wernery & Kaaden, 2002).

There are not enough studies about the frequency of camel dermatophytes in Iran. In a study between 1994 and 1998, 22% of camel specimens from different areas of the country were infected with dermatophytosis (Khosravi & Mahmoudi, 2003). In another study during 2003–2013, 74% of camel samples belonging to warm and humid regions of Iran were positive for *Trichophyton verrucosum* (Shokri & Khosravi, 2016). Routine and regular inspection of camels, isolation of infected camels, disinfection of contaminated stables, vaccination and improved hygiene are useful measured to manage dermatophytosis in camels.

## 4 DISCUSSION

There are 171,500 dromedary camels distributed across 22 out of 31 provinces of Iran, 70% of which live in Sistan and Baluchestan and Khorasan provinces in the east of Iran. Some camels are smuggled from neighbouring countries, particularly Afghanistan and Pakistan, to Iran with no surveillance and monitoring systems, or maybe the systems are neglected for such animals. Thereby, there is a potential threat of bringing in new non‐endemic pathogenic organisms into Iran that have not already seen in Iranian camels; therefore, such trade and importation must be limited as much as possible (Mogghadass, 2012; Moghaddas, 2012 2012).

Reported camel zoonotic diseases in Iran can be divided into two major categories. The first group is devoted to the diseases that are rare in Iran and received limited studies including plague, MERS‐CoV and CCHF. More attention should be paid to these diseases as they are neglected most of the time. The second category is related to the diseases that are common in Iran but received very little attention in the majority of studies (Yavari, 2013). These should be considered the most important zoonotic diseases of Iranian camels, including leptospirosis, Q fever, brucellosis, campylobacteriosis, tuberculosis, pasteurellosis, salmonellosis, *E. coli*, listeriosis, rabies, camelpox, echinococcosis, cryptosporidiosis, toxoplasmosis and dermatophytosis. Specific strategies, therefore, should be planned to have a better prevention and control program for each of the above diseases in the country.

The prevention and control of mentioned diseases are dependent on having enough information on the infectious agent, occurrence, transmission method, incubation period, immunity and control programs.

If the mode of transmission is known, precautions can be put in place to prevent outbreaks of diseases. Some disease‐control interventions are directed towards the mode of transmission including direct contact, droplets, a vector such as a tick and food‐borne or air‐borne routes. Diseases such as leptospirosis, brucellosis, salmonellosis, pasteurellosis, campylobacteriosis, MERS‐CoV, CCHF, cryptosporidiosis and dermatophytosis can be transmitted through direct contact with animal tissues or fluids including nasal secretion, saliva, urine, placenta and faeces. In the case of CCHF, the disease can also be transmitted through direct contact with camel fresh meat or the blood of viraemic animals (Kloos & Berhane, 2006). Consumption of raw or half‐raw milk and other unpasteurized dairy products can transmit plague, Q fever, listeriosis, brucellosis, campylobacteriosis and tuberculosis from camel to human (Kloos & Berhane, 2006). Plague, listeriosis and campylobacteriosis can also be transmitted from camel to human by consumption of undercooked meat. Leptospirosis, campylobacteriosis, *E*. *coli* infections, salmonellosis, cryptosporidiosis can be transmitted by the ingestion of food or water contaminated with the urine or faeces of infected camels. Diseases such as tuberculosis and Q fever can be transmitted through contaminated aerosols, fluids aerosolized from an animal to a person (e.g. sneezing or coughing), or by inhaled aerosolized materials. Rabies and pasteurellosis can spread by infected, diseased or source animal bites and scratches. Plague, camelpox, MERS‐CoV and CCHF are transmitted by an infected arthropod vector (e.g. fleas or ticks); therefore, these agents can be controlled by appropriate anti‐ectoparasite drugs (Kloos & Berhane, 2006; Murphy, 2008).

Several major steps need to be taken in order to prevent and control camel zoonotic diseases. The most valuable preventive step is vaccination, not only for the protection of individual animal but also to build up a level of population immunity that suffices to break transmission. It seems that vaccination can be an effective way to control diseases such as camelpox and brucellosis among camels.

Because camel is one of the hosts and zoonotic agents circulating in other species (except for MERS‐CoV and camelpox), screening and control of other animals should be followed as well, in addition to monitoring these agents in camels. Moreover, the birth certificate of camels and more accurate monitoring of their movements should be seriously considered to have a better surveillance program among Iranian camels.

There should also be full supervision to prevent the slaughter of camels at homes or public places. Camels should be slaughtered only in standard slaughterhouses under the control and supervision of a veterinarian, who uses personal protective equipment and is aware of endemic diseases in camels and their mode of transmission to human.

Camel meat and dairy products must be purchased from authorized veterinary locations, and consumption of raw or undercooked camel's meat and unpasteurized dairy products should be avoided. Camel and its by‐products (fur, wool and uncleaned head and trotter) should be under the control of veterinary systems during shipment.

## 5 CONCLUSIONS

In this study, 19 reported camel zoonotic diseases and infections in Iran were reviewed and outlined with referring to reported regions. It seems that camel infections are neglected in Iranian academic researches. Future studies, therefore, should address this topic to determine the exact impacts of camel diseases on public health in Iran.

Ministry of Health, Ministry of Agriculture and Environmental Organization must pay more attention to echinococcosis, brucellosis, salmonellosis and MERS‐COV in order to control these diseases and infections in camels and prevent their transmission to humans. Authorities should focus on herd health management including extended screening, vaccination, and training and paying more attention to water and food safety. As some of camel zoonotic diseases are imported from other countries, monitoring should be seriously considered on the birth certificate of the camels and their movements.

## 6 ETHICS STATEMENT

The authors confirm that the ethical policies of the journal, as noted on the journal's author guidelines page, have been adhered to. No ethical approval was required as this is a review article with no original research data

## CONFLICT OF INTEREST

The authors declare that they have no conflict of interest.
